# Burnout in Assisted Reproduction Professionals: The Influence of Stressors in the Workplace

**DOI:** 10.3390/healthcare12212136

**Published:** 2024-10-26

**Authors:** Raquel Urteaga, Amelia Díaz

**Affiliations:** Faculty of Psychology, University of Valencia, 46010 Valencia, Spain; raurgar@alumni.uv.es

**Keywords:** burnout, assisted reproduction professionals, workplace stressors, emotional exhaustion, depersonalization, personal accomplishment, working under pressure

## Abstract

Background/Objectives: Most of the research in assisted reproduction has focused on the stressful situation experienced by women or couples looking for a desired pregnancy; however, the stress experienced by assisted reproduction professional teams is seldom studied. The present study aims to evaluate burnout and its relationship with workplace stressors among assisted reproduction professionals. Methods: A cross-sectional design was used to conduct an online self-assessment national survey, sent to all members of the Spanish Association for Fertility. The questionnaire contained sociodemographic and occupational questions about stressors in the workplace and the Maslach Burnout Inventory (MBI-HSS) to assess the three subscales of burnout: emotional exhaustion, depersonalization and personal accomplishment. Results: The percentages showing high emotional exhaustion and depersonalization in the whole sample were 41.8% and 43.2%, respectively. Additionally, low personal accomplishment was displayed in 42.6% of the respondents. Embryologists stand out for presenting the highest percentages of burnout (emotional exhaustion = 72.1%; depersonalization = 48.1%; low personal accomplishment = 48.1%), whilst psychologists showed the lowest percentages in high emotional exhaustion (32.3%) and depersonalization (24%), and gynecologists in low personal accomplishment (28.5%). Working under pressure was the stressor most experienced by the sample (76.6%) and the one that better predicted the three subscales of burnout. Conclusions: This study highlights the close relationship between high levels of burnout and workplace stressors and shows the need to reduce workplace stressors to improve the well-being of professionals working in assisted reproduction, and, consequently, that of the patients they look after.

## 1. Introduction

Burnout is described as an occupational phenomenon produced by chronic stress in the workplace [1]. Previously, in 1974, Freudenberger [2] defined burnout as a psychological state characterized by fatigue and frustration due to unsuccessful professional expectations usual in care service workers. This exhaustion in the performance of the work resulted in a loss of motivation and in some cases aggressive behavior towards the care recipients. Two years later, Maslach [3] summarized the definition, stating that burnout is a gradual process where workers present three characteristics related to their work. Firstly, emotional exhaustion (EE), a feeling of exhaustion, fatigue, weariness, showing an inadaptation to the work environment and a lack of emotional energy to cope with the day-to-day tasks. In second place is depersonalization (D) or cynicism, described as a negative, unconcerned, detached or indifferent attitude towards the work or users. Finally, the third burnout dimension is a lack of personal accomplishment (PA) and consists of a perceived loss of sense of professional efficacy, with a tendency to evaluate negatively both the work performance and their own abilities and capacities to do it.

Different theories have explained what aspects propitiate, trigger, and/or maintain workers suffering from burnout, some of them, such as the social cognitive theory, focus on the worker’s individual variables, highlighting self-efficacy, self-confidence and self-concept [4], but theories with a higher impact [1] are those based on organizational or workplace stressors such as the organizational and the demands–resources theories. The organizational theory postulates that burnout is produced by organizational and workplace stressors such as working under high emotional or physical pressure, work overload, and ambiguity in the role producing conflicts when working in a team [5,6], whereas the demands–resources theory highlights the imbalance between the work demands and resources, where common demands are work overload, emotional labor, time pressure, or interpersonal conflicts [7].

On the other hand, Assisted Reproduction (AR) professionals form interdisciplinary teams made up of embryologists and lab technicians, clinicians, including gynecologists/obstetricians, andrologists, nurses and nurse’s assistants, and counsellors, normally psychologists [8]. When revising the literature in the field of AR in association with stress, most of it relates to the stress experienced by the women or couples who wanted a pregnancy. The number of papers studying the stress suffered by the professionals in their workplace, when performing those procedures, is scant in comparison and tends to concentrate on stress and burnout in specific AR professions, rather than on the AR teams. For example, it is possible to find papers about the stress experienced by embryologists, gynecologists, nurses or psychologists, but separately, or as general medical or health professionals and not as members of an AR team. Most publications focus on the stressful situations experienced by the embryologists, as the most characteristic component of the AR teams and, unfortunately, some of the published research relates to the errors or mistakes these specialists seldom make [9].

There are, however, a few exceptions to this trend. Based on the causes of discontinuation or drop out of AR by couples, Boivin et al. [10] proposed an “Integrated Approach to Fertility Care” aimed at reducing AR professionals’ burden in their day-to-day involvement with patients. Although the model included three important factors in the patient’s discontinuation: patient, AR professionals and the treatment, it is important that when considering AR professionals as a cause of patient drop out, the model identifies the factors that AR professionals associate with burden: stressful situations, inadequate coordination between them, depersonalized process, negative attitudes, work overload due to bureaucratic procedures, and negative staff–patient interaction due to lack of time, poor listening skills, low empathy, etc. Another exception is the qualitative study of Fanchim et al. [11] in infertility care providers in two Italian AR clinics, where embryologists, gynecologists, psychologists, and nurses were included. With the use of semi-structured interviews with 23 AR professionals, this study identified three stressful themes in the work of AR professionals. The first and most important one seems to be the communication between AR professionals and patients, in this case, the patients were mostly women due to the lower involvement of men in the process. These professionals described some women with intense negative feelings such as anxiety, frustration, pessimism, and in some cases, depression. From the point of view of the AR professionals, women with this attitude tended to blame the doctors for the lack of success in the treatment, expressing a lack of trust in them, without considering other factors, such as intrinsic rates of success, pharmacological effects, risk of surgical interventions or patient’s age. Dealing with complicated or difficult patients is a common complaint among physicians including AR professionals [12]. A second source of stress in AR teams came from organizational factors. Working in a team that has many different roles, but a common goal could lead to stressful situations due to members’ personalities, skills and coping styles. These variables become especially important when the goal is not achieved, and the team must deal with sensitive or difficult patients. Finally, the third theme in AR professionals is related to the type of work these professionals perform, supporting a high responsibility with no room for errors and where the role of embryologists is one of relevance and vital importance.

Embryologists work with cells, but not normal cells. What they help to create represents a hypothetical future individual, and that is where the great responsibility comes from, and the reason they work not alone but teamed with other embryologists or lab technicians. Avoiding errors is a priority for them. The work they perform requires intense concentration, where overload and complex techniques could produce exhaustion and burnout [13]. Some studies have shown a high proportion of embryologists suffering from work-related musculoskeletal disorders, and mental health problems [14]. Specifically in Spain, the percentages of embryologists with musculoskeletal disorders were 45.3% and 27.8% suffered from stress and mental health problems [15]. A stressful situation experienced by these professionals concerns the high expectations that patients have and the difficulty dealing with them when an unsuccessful intervention happens and they must give explanations regarding oocytes and embryos, in a language that patients can understand [16]. These professionals reported high levels of burnout; however, the levels depend on the country studied and the time when the study was performed. López-Lería et al. [15], in one of the first studies, evaluated burnout in 2014 in these Spanish professionals, finding that the percentages of embryologists showing EE was 20%, D was 15% and low PA was 10.8%. However, Murphy et al. [17], ten years later, presented results in the UK and the USA that, depending on the specific subscale and country, double, triple or quadruple Lopez-Leiria’s results [15] (for the UK, EE = 59%, D = 43% and low PA = 15%; for the USA, EE = 62%, D = 60% and low PA = 28%).

Nurses working in AR services showed significantly high levels of burnout in the three subscales, emotional exhaustion, depersonalization and low personal accomplishment [18]. Kilstein pointed out that, because of being in a more close and personal relationship with the patient than embryologists, nurses experienced a very stressful situation and significant emotional responses and, in the event of failure of the AR treatment, the consequence of the difficulty of dealing with patients’ questions in this unfortunate context [19]. When nurses were compared with doctors and lab technicians in private AR centers, nurses had higher occupational stress index scores compared with the others, associated with long working hours, the stressful working conditions in AR centers, and the high emotional burden of dealing with infertile couples [20].

Gynecologists are not free from stressful situations and burnout either. Findings coming from Moradi et al. [21] and Thorsen et al. [22] presented a prevalence of 44–56% among them, especially high in the three subscales of burnout: emotional exhaustion (72%), low personal accomplishment (74%) and depersonalization (43%). In the case of younger residents working in the gynecology service, half of them (50%) reported high emotional exhaustion and depersonalization [23].

Finally, the role of the psychologist in AC teams is to support patients and help them, with efficient coping skills, to deal with the situations they could face in AR treatment, from the infertility diagnosis to the emotional and stressful situations they will experience in AR treatment [24]. In Spain, most AR clinics include psychologists, but not all clinics around the world provide psychological support for patients in their AR teams; therefore, several AR professional societies claim for the inclusion of mental health professionals in AR teams [25]. Mental health providers also experienced burnout [26], but in AR services they provide great benefit to patients, as shown in a study of 7456 female fertility patients that reported greater positive treatment experiences when fertility clinics offered onsite mental health counselling [27]. An added advantage of having the psychological service offered in the AR clinics is that this service could be also used by the medical staff working with difficult patients, coping with other work-related stress, and sharing difficult news. However, the inclusion of mental health professionals presents its own difficulties. In a qualitative analysis performed on a sample of 28 Italian AR psychologists, these professionals expressed loneliness and lack of integration within the medical field [24]. A previous study highlighted the difficulty that psychologists in AR have implementing a multidisciplinary model whose users were not only patients but also all professionals working in the team [28]. It seems that psychologists are not only aware of the psychological needs present in ARpatients but also of those of their teammates.

In addition to the type of AR-related professions, it is necessary to consider some demographic variables, such as age and gender, that play a role in the assessment of burnout. There are studies show that among health workers, women and younger workers face higher scores in burnout than men and older ones [29,30,31,32]. Among the factors contributing to explaining gender differences are that women perceived higher workload, dedicated more time per patient, received fewer resources, reported less control over their workload and schedules [30], presented lower rates of professional fulfillment [24] and worked a greater number of hours in clinical work [32]. This gender difference is also present in professionals working in obstetrics and gynecology clinics, where female gynecologists and nurses showed higher levels of burnout than male ones [33,34,35,36]. Regarding age, studies performed involving nurses and gynecologists showed that older professionals presented lower burnout levels than younger ones [34,35,36].

From the accumulated literature on AR professionals, only two reports studied AR teams as a whole. The first of these reports analyzed the causes of drop out by couples [10], where the central point was the couples attempting a pregnancy and the causes for them dropping out of the process, whilst the second, the qualitative research of Fanchim et al. [11] with a small sample of embryologists, gynecologists, psychologists, and nurses, used semi-structured interviews. The remaining studies looked at each one of the AR professions separately [12,13,14,15,16,17,18,19,20,21,22,23,24,25,26,27,28]. Clearly then, there is a gap in the research on AR that can only be filled with additional studies on AR teams as a whole, with a good representation of all AR professionals and including a separate analysis of each profession in relation to burnout and the role of workplace stressors.

The general aim of this work is to measure the stress in AR teams. Specifically, the objective of this research is to evaluate burnout in embryologists, gynecologists, psychologists, nurses and nurse assistants and study its relationship with workplace stressors. The hypotheses derived from the revised literature in the fields are as follows:

1. AR professionals will present moderate-to-high levels of burnout in their workplace, this being reflected in the subscales’ means placing most of the AR professionals in the moderate and high categories.
2. Female professionals will show higher burnout than male ones.
3. Older professionals will present lower burnout than younger ones.
4. Embryologists, gynecologists, nurses and nurse assistants will present higher percentages in the high level of burnout subscales whereas psychologists will present the lowest percentages.
5. The demographic variables and stressors measured in this study will significantly predict the three subscales of burnout in the sample.

## 2. Materials and Methods

### 2.1. Procedure, Participants and Design

An online survey was created on the Survio platform and distributed through the Spanish Society of Fertility newsletter to all its members (n = 1575). The society is composed of different professionals from the field of AR. The newsletter contained a description of the research and a link that gave access to it. It was stated in the newsletter that anyone answering the survey understands the purpose, objectives, has the necessary information on the research and consents to participate. The participation was voluntary, anonymous and no reward was obtained for participation. The national survey contained demographic questions such as age and gender, questions about usual stressors in their workplace and the Maslach Burnout Inventory. Of the 1575 members of the Spanish Society of Fertility, 408 AR professionals responded to the online survey, that is 25.90% of their members, although eight did not finish the survey and were removed. The survey was completed between 15 November and 15 December 2023. The professionals that responded to all questions were embryologists (n = 104), gynecologists (n = 116), psychologists (n = 31), nurses (n = 74), nurse assistants (n = 55), andrologists (n = 7), geneticists (n = 4), nutritionists (n = 2) and lab technicians (n = 7). Following the central limit theorem [37] that assumes the normality in the distribution of variables begins at the point of 30 participants, and the use of parametric statistics that recommended a minimum number of participants per group of 30 [38], andrologists, geneticists, nutritionists and lab technicians were removed from all analyses in this study due to the low number of participants, and the consequent loss of representativeness. Therefore, the final sample was 380 AR respondents with an age mean of 42.52 years (SD = 10.22 years) and the age range was 21–65. In relation to gender, 57 (15.0) were men, 321 (84.5%) were women and 2 (0,5%) reported “other” gender. The design used was cross-sectional and the permission to conduct this research was obtained from both the Board of Directors of the Spanish Society of Fertility and the Ethical Committee for Scientific Research of the authors’ University (Code: 3468621).

### 2.2. Measures

Maslach Burnout Inventory (MBI-HSS) [39] validated in Spanish professionals [40] was used to obtain burnout measures. The inventory contains 22 items with the 0–7 Likert scale (0 = never, 1 = a few times a year or less, 2 = once a month or less, 3 = a few times a month, 4 = once a week, 5 = a few times a week, and 6 = every day). The items are distributed across the three subscales of emotional exhaustion, depersonalization and personal accomplishment. Higher scores in EE and D, but low in PA indicate higher level of burnout. The subscale scores can be calculated using two methods. The first method sums the item scores for each subscale, and the second method distributes respondents in the low, moderate and high categories for each subscale, using the following cut-points: emotional exhaustion (low ≤ 18; moderate 19–26; high ≥ 27), depersonalization (low ≤ 5; moderate 6–9; high ≥ 10) and personal accomplishment (low ≤ 33; moderate 34–39<; high ≥ 40) [41]. The reliability obtained in this study performing Cronbach’s α (EE = 0.90; D = 0.71 and AP = 0.73) was adequate.

The survey included questions about demographic variables such as gender and age, and questions about stressors that were usual in their workplace such as: 1. working under pressure, 2. overloaded schedule, 3. lack of professional recognition, 4. communication difficulties between the team members, 5. management of complicated patients and 6. breaking bad news to patients, all of them answered in a Yes/No format.

### 2.3. Statistical Analysis

Previously to perform any further analysis, skewness and kurtosis of burnout subscales were analyzed to test their adaptation to normal curve, and reliability was obtained performing Cronbach’α for the three burnout subscales. Next, burnout was analyzed using the means, standard deviations and percentages of AR professionals in the categories of low, moderate and high for the burnout subscales in the whole sample. In second place, gender and differences analyses were performed to know if men and women or older and younger AR professionals show differences in burnout subscales. Thirdly, descriptive analysis was performed to obtain means and standard deviations in the three burnout subscales, and percentages of high EE, high D and low PA for the five types of AR professionals studied: embryologists, gynecologists, psychologists, nurses and nurse assistants. Additionally, percentages of each type of AR professional experiencing the six types of stressors were obtained. Finally, a hierarchical regression for each of the three burnout subscales was performed, in the first model with the demographical variables, gender and age, and in the second model with the addition of the six workplace stressors to know the role of demographical variables and stressors for predicting EE, D and PA. Analyses were performed using the Software IBM SPSS Statistics for Windows, version 28 (IBM Corp., Armonk, NY, USA).

## 3. Results

Table 1 shows firstly that the subscales of burnout emotional exhaustion, depersonalization and personal accomplishment presented a normal distribution since the skewness and kurtosis values were not above −2 and below +2 [42] and close to zero [43]. Secondly, the sample’s means in the three subscales of burnout could be categorized in the moderate level but looking at the percentages of AR professionals in the three categories, low, moderate and high in these subscales, 41.8% of the sample presented high emotional exhaustion, 43.2% showed high depersonalization and 42.6% scored low in personal accomplishment in the performance of their jobs.

Gender differences were not significant between men and women in the three burnout subscales: emotional exhaustion men’s mean = 23.65 (SD = 14.91); women’s mean = 23.94 (SD = 12.36); t (376) = −0.16; *p* = 0.874; depersonalization men’s mean = 9.23 (SD = 7.08); women’s mean = 8.56 (SD = 5.90); t (376) = 0.77; *p* = 0.444; and personal accomplishment men’s mean = 34.71 (SD = 6.65); women’s mean = 34.40 (SD = 6.97); t (376) = 0.30; *p* = 0.761. Age showed a unique, significant difference in the subscale of personal accomplishment, F(32,347) = 2.09, *p* = 0.044, partial η^2^ = 0.038, where younger professionals scored lower than older ones. The lowest score in personal accomplishment was for the 21-year-old professionals (mean = 25.00) and the higher scores (mean ≥ 35.00) were shown by professionals older than 50 years. Age did not present significant differences for emotional exhaustion, F(32,347) = 0.82, *p* = 0.568, partial η^2^ = 0.015, and depersonalization F(32,347) = 1.74, *p* = 0.100, partial η^2^ = 0.032.

Using a more specific approach, looking at the different professionals working in AR (see Table 2), embryologists showed the highest scores in emotional exhaustion (mean = 25.64) whilst psychologists presented the lowest scores in the same subscale (mean = 19.55). However, the Bonferroni post hoc analysis did not show significant differences between the five professions compared. The results are similar in the depersonalization subscale, with embryologists being the professionals with the highest score (mean = 10.25) and the psychologists with the lowest one (mean = 6.00), with the Bonferroni post hoc analysis showing two significant differences between embryologists and psychologists (*p* = 0.006) and embryologists and nurse assistants (*p* = 0.018). The reverse pattern was found in the personal accomplishment subscale, where embryologists presented the lowest score (mean = 32.49) and psychologists had the highest one (mean = 37.61). The post hoc Bonferroni comparison showed two significant differences between the embryologists and the gynecologists (*p* = 0.005) and the embryologists and the psychologists (*p* = 0.003). The percentages of specific professionals with high levels of emotional exhaustion and depersonalization and low level of personal accomplishment show similar results using the means; the embryologists clearly present higher signs of burnout in the form of emotional exhaustion (72.1%), depersonalization (48.1%) and low personal accomplishment (48.1%) when compared with other professionals working in AR. Almost three-thirds of them show emotional exhaustion, and half present depersonalization and low personal accomplishment.

Six stressors were analyzed, both in the whole of the sample and in the different professions in AR (see Table 3). Performing the work under pressure is the stressor most suffered in AR work, with over three-quarters of the sample working in this situation (76.6%). Only the psychologists presented lower percentages in this stressor (45%), but still close to half of them; embryologists and gynecologists were over 80%, and nurses and nurse assistants were over 70%. As the percentages show, the effect of this stressor on the different AR professionals is significantly different (Χ^2^ = 22.36; *p* < 0.001).

Around 45–55% of the sample in this study reported suffering from stressors such as overloaded schedules, communication difficulties between the team members and management of difficult patients. An overloaded schedule seems to be shared by all five types of professionals in a similar way, with psychologists being the professionals suffering this stressor at the lowest level (45.2%) and nurses at the highest (56.8%). There were no significant differences between the percentages of the five AR professions (Χ^2^ = 1.44; *p* = 0.838). Communication difficulties between the team members are more present in embryologists (49%) and gynecologists (47.4%), which is almost half of them, with psychologists again being the least affected (32.3%). No significant differences in the percentages for this stressor were found between the AR professions (Χ^2^ = 3.31; *p* = 0.507). The management of complicated patients is an important stressor mainly for nurses (71.6%), nurse assistants (61.8%) and embryologists (61.5%), with gynecologists being the least affected (29.3%). Significant differences were found between the percentages of AR types of professionals for this stressor (Χ^2^ = 41.25; *p* < 0.001).

Finally, the stressors of lack of professional recognition and breaking bad news to patients seem to affect a lower percentage of the whole sample (31.1%). Lack of professional recognition is reported mainly by gynecologists, with the highest percentage (45.7%) whilst embryologists and psychologists are the least influenced by this stressor, with percentages of 17.3% and 16.1%, respectively. This stressor presents significant differences between the percentages of the AR professions (Χ^2^ = 27.71; *p* < 0.001). Breaking bad news to patients is present in nurses (45.9%), embryologists (43.3%), psychologists (41.9%) and nurse assistants (40%) with similar percentages, whereas gynecologists are less influenced (23%). Significant differences between the percentages in the five professions were found (Χ^2^ = 14.19; *p* = 0.007).

Three hierarchical regressions were performed for each burnout subscale with the contribution of gender and age variables in the first model and both demographic variables and the six stressors in the second model. The results are presented in Table 4. Model 1 shows that age predicts depersonalization (D), being higher in younger professionals than older ones, although the change in R^2^ is not significant (*p* = 0.074). Additionally, age predicts personal accomplishment showing a significant change in R^2^ (*p* = 0.20), where older professionals show more personal accomplishment than younger ones. The introduction of the six stressors in Model 2 makes the significance of age disappear. When the six stressors were introduced, the change in R^2^ was highly significant for the three subscales, EE (B = 26.78, *p* ≤ 0.001), D (B = 6.30, *p* ≤ 0.001) and PA (B = 6.99, *p* ≤ 0.001), highlighting that working under pressure is the only stressor significantly affecting the three burnout subscales, EE (B = 12.19, *p* ≤ 0.001), D (B = 3.39, *p* ≤ 0.001) and PA (B = −3.14, *p* ≤ 0.001). Emotional exhaustion is also affected significantly by all remaining stressors except for an overloaded schedule. Depersonalization is also predicted significantly by communication difficulties between the team members. And lack of personal accomplishment is also predicted by management of complicated patients and breaking bad news to the patients.

## 4. Discussion

The aim of the present study was to measure stress and burnout in AR teams including many different professions at the national level in Spain. However, in the case of andrologists, geneticists, lab technicians and nutritionists the number of respondents was lower than that required for statistical analysis. The low number of responses from these professionals reflects the fact that not all AR teams include them. Accordingly, this study included the core of professions that are present in AR teams in Spain, that is, embryologists, gynecologists, psychologists, nurses and nurse assistants.

The number of published studies focusing on AR professional teams is scarce, with a low number of participants and in some instances not really having the AR teams as the main research focus, except in the context of couples dropping out of the AR process [10,11]. Our study intends to contribute to filling the research gap in the assessment of burnout and workplace stressors in AR teams by looking at AR teams as an item in the analysis of burnout and workplace stressors. This approach allows a deeper analysis of specific stressors affecting all members of the AR teams, without losing the separate analysis of each individual profession involved in AR.

The results showed the existence of moderate burnout levels in our sample, considering the means in each burnout subscale, EE, D and low PA, but the percentage of AR professionals in the high EE and D, and low in PA subscales was over 40%, and over 63% taking together moderate and high percentages. That means that one in every three AR professionals was having serious problems related to burnout. Therefore, considering the results of all participants, the first hypothesis stating that AR professionals will present moderate-to-high levels of burnout in their workplace is confirmed. Although to our knowledge there is not a study with data on burnout in AR teams, these results confirm similar results found in physicians [31,43], and separately in AR embryologists [15,16], gynecologists [21,22,23], nurses [18] and psychologists [26] in all cases showing high burnout levels.

The second and third hypotheses are based on the influence of the demographic variables gender and age in burnout. The hypotheses proposed that women and young professionals will experience more burnout than men and older professionals. Although previous studies found higher levels of burnout in women working in health professions than in men [29,30,31,32,33,34,35,36], our results show no significant differences between both genders. The percentage of women working in AR teams is very high, and it is a field where stress and burnout levels are high. Our results suggest that burnout would be more affected by the role played in the AR team than by gender. The role of age seems to give some advantage to older professionals who present a lower level of burnout than younger ones [34,35,36]. This study only confirmed this statement in the subscale of personal accomplishment, with older professionals presenting higher PA than younger ones. Age seems to help AR professionals to better deal with patients’ problems and give a better understanding and the feeling of having accomplished worthwhile things through their work.

Psychologists as providers of mental health care for women and couples in AR treatments are in the position to implement psychological treatments that could help to face stressful situations [24]. They have the formation and the learned skills to deal with psychological problems, but they can also experience burnout in their workplaces [26]. However, they are in a more favorable position and the burnout should be lower than other AR professionals who do not have the psychological formation they have. The medical professionals in the study, embryologists, gynecologists, nurses and nurse assistants could be more susceptible to burnout and the effects of other stressors in the workplace. Again, hypothesis four is confirmed, with psychologists showing the lowest levels in the burnout subscales, whereas the medical professionals obtained higher scores. What is especially remarkable from our results is the high percentages of embryologists showing high emotional exhaustion, almost three out of four, and high depersonalization and low personal accomplishment, in both cases almost half of them. These results show a clear picture of where embryologists stand out from the rest of the professionals in AR teams, having the highest levels of burnout. The level of EE is higher than any other found in embryologists in studies performed in Spain, where Lopez-Lería et al. [15] found 20% in 2014, and higher than those found by Murphy et al. [17] in the UK (59%) and the USA (62%) in 2024. The percentage of embryologists with low PA (48.1%) is also higher than those found in the cited studies: López-Lería et al. [15] found 10.8% whereas Murphy et al. [17] found 15% in the UK and 28% in the USA. Finally, the percentage of high D is higher (48.1%) than those found by Lopez Lería et al. [15] (15%) but just between the percentages found in the UK (43%) and the USA (60%) [10]. The increase in burnout presented by embryologists seems to be more related to the increased workload derived from the increase in the number of people in AR treatment nowadays [44] than to the specific country where the study was performed.

The role of the six stressors evaluated in the AR teams in our study is relevant, especially the stressor of working under pressure, experienced by over three-quarters of the professionals that answered the questionnaires, mainly the embryologists and gynecologists with four out of five, and nurses and nurse assistants with almost three out of four. The responsibility, the work overload and the limited time to perform the tasks could be related to these high percentages of medical staff working under pressure [13,20]. Psychologists are the AR professionals presenting the lowest percentages, but the fact that almost half of them also report working under pressure cannot be ignored. Overloaded schedules and the management of difficult patients are the following stressors in order of high percentages presented by the AR professionals, with percentages over 50%, meaning that over half of the AR professionals who answered the questionnaires had experienced these stressors. Whereas in the overloaded schedule stressor, the percentages of the five types of AR professionals are similar, in the case of the stressor management of difficult patients, embryologists, nurses and nurse assistants show the highest percentages, whilst gynecologists show the lowest. Likewise, in the stressor breaking bad news to the patients, a low percentage of gynecologists reported this stressor compared to other professionals in the AR team, and these low percentages shown by gynecologists could be related to the previous stressor: management of difficult patients. Dealing with the bad news of unsuccessful AR treatment is a stressful situation that could lead the AR professionals to perceive the patient as “difficult” [12]. Psychologists also have an important role in this sad context, helping patients to cope with the situation, but this is their specific role and they are trained for it. Finally, gynecologists are the professionals less affected according to the results of this study. In general, these results confirm those by other authors where embryologists [16] and nurses [19,20] are placed in a stressful situation when explaining failure in AR interventions in a language that patients can understand.

Two stressors presented lower percentages compared with the four stressors discussed above: lack of professional recognition and communication difficulties between the team members. The lack of professional recognition is more often reported by gynecologists, with psychologists having the lowest percentages, whereas communication difficulties between the team members showed similar percentages for all team members, with embryologists reporting the highest percentages and psychologists the lowest. The role of this stressor was highlighted by Fanchim et al. [11], giving an important role to the fluidity of communications between professionals and patients.

The psychologists in our study present a different picture to the rest of the professionals in the AR teams, showing lower levels of burnout and being affected to a lower degree by stressors. This result echoes those of Fanchim et al. [11] and Di Trani et al. [24], who highlighted a feeling of loneliness and a lack of integration of psychologists with the medical AR professionals, forcing psychologists to play a more independent role and allowing them to be less affected by the stress. On the other hand, embryologists are at the top, both in burnout symptoms and in being highly affected by workplace stressors, mainly working under pressure. These results mostly confirmed the fourth hypothesis in our study. A closer and deeper analysis of the reasons why embryologists are the AR professionals suffering the highest level of burnout among all the professions involved in the process is clearly necessary. Two factors tentatively could explain this result: the increased use of AR worldwide in the last decades, with the consequent work overload [44] and the increased level of bureaucratic work embryologists must perform according to their country’s legislation [45].

The prediction of the three subscales of burnout, EE, D and PA, showed a common stressor, working under pressure, significant in all cases. Emotional exhaustion is predicted basically by all stressors with the single exception of an overloaded schedule; therefore, lowering this burnout dimension involves control and decreases most of the stressors evaluated. The second dimension, depersonalization or the presence of cynical attitudes is also related to communication difficulties between the team members, so better communication skills and the decrease in pressure in the work could improve these negative attitudes. Finally, personal accomplishment increases when the pressure in the work is lower and the relationship between professionals and patients is better, especially in the case of AR unsuccessful interventions. A final variable to consider when predicting burnout is age. We confirmed that older AR professionals showed higher personal accomplishment than younger ones, although the role of age in predicting burnout disappears when the stressors are introduced in the regressions. The fifth hypothesis is only partially confirmed because not all stressors significantly predicted the three burnout subscales. However, at a low or high level, all stressors studied affected the AR professionals of the sample, hence the need to lower their effect on these professionals. The inclusion of an “Integrated Approach to Fertility Care”, focused on the patients, AR professionals and treatment, as Boivin et al. [10] proposed, could improve the well-being of patients and AR professionals and reduce the burden on AR professionals, helping them to cope with stressful situations, increasing the coordination between them, creating an AR process that is more personal and humanized, with more positive attitudes and an improved patient–professional relationship.

The main aim of this research, the evaluation of burnout and workplace stressors in AR teams, was achieved, with our results showing a picture where the burnout levels are higher than what was expected for the AR teams as a whole sand, at the same time, highlighting the specific case of the embryologist. Burnout levels in our study are higher than those found in previous studies [15,17], and this may reflect the increased demand for AR in the last few decades, with the corresponding overload of the AR teams [44,45], making the need for changes evident. Most burnout interventions are based on two factors, the individual worker and the workplace [46]. Intervention at the individual level involves changes that the person must undertake to increase his/her well-being, strengthening both physical and psychological resilience, with mindfulness training among the recommended interventions [47]. Workplace intervention is based on workplace strategies that eliminate and modify work stressors. Our study confirmed the role of workplace stressors, highlighting the role of working under pressure, work overload, lack of professional recognition, communication difficulties between the team members, management of complicated patients, and breaking bad news to patients. Organizational intervention should focus on lowering the pressure (on time, responsibility or tasks) these professionals are feeling by reducing the level of work required, increasing the professional recognition in AR teams, and training them in communication skills to improve the relationships between the AR team members, and the communication and relationship of the team members with the patients.

### Limitations and Future Research Directions

This study presents several limitations. The number of participants could be higher, especially in the case of AR professionals such as andrologists, geneticists, nutritionists, and lab technicians, who have not been included in this study due to the low number of participants. The design is cross-sectional descriptive; therefore, the information is relevant to the time the study was performed and no causal inference can be made. In addition, all measurements were obtained through self-reports with the bias that this type of measure includes, such as social desirability, acquiescence, difficulties being truthful or difficulties in introspection ability. The variables measured are limited to demographic information, burnout and workplace stressors; the addition of other variables such as personality traits could provide valuable insights into risk factors for burnout, such as participants’ level of neuroticism. Although the sample is representative, being a nationwide survey, the results should be restricted to Spanish AR professionals.

Future research in the area should try to address gender imbalance. Although most professionals in the health field are women, an effort to increase the number of men in the studies should be made. For example, it could be very interesting to know the role of gender in each AR profession. Likewise, longitudinal studies, with repeated measures could shed more light on how burnout evolves over time in AR professionals. Also, the inclusion of more types of variables could improve the results: sociodemographics such as marital status, number of children, salary; workplace variables including more stressors not studied here; and psychological variables such as social support, coping strategies, etc. Finally, an international survey including burnout measures and stressors in AR professionals in other countries could enrich the results found in Spain. Finally, the use of standardized protocols for each profession in AR will help to manage the work and specific stressors that these professionals face in the workplace.

## 5. Conclusions

This study evaluated burnout and workplace stressors among AR professionals, presenting data for both the AR team as a whole and the five professions assessed. We can conclude that as a team, one in every three AR professionals experienced moderate-to-high levels of burnout symptoms, with embryologists being the ones that presented the highest levels of burnout and psychologists the lowest. The burnout levels were closely associated with all the workplace stressors measured in this work, but one of them, working under pressure, affected the three main symptoms of burnout: emotional exhaustion, depersonalization and personal accomplishment. Our study has shown the role workplace stressors play in the burnout presented by AR teams, and points to the need to make the necessary changes both at the individual level but mainly at the organizational one, to implement strategies or interventions that eliminate or modify these stressors, reducing burnout and increasing the wellbeing of AR professionals, something that will, in the end, benefit the patients.

## Figures and Tables

**Table 1:** Skewness, kurtosis, means, standard deviations and categorization of Maslach Burnout Inventory subscales in the whole sample.

				Low	Moderate	High
	Skewness	Kurtosis	Mean (SD)	% (n)	% (n)	% (n)
Emotional Exhaustion	0.20	−0.76	24.01 (12.81)	35.5 (135)	22.6 (86)	41.8 (159)
Depersonalization	0.55	−0.31	8.72 (6.13)	32.9 (125)	23.9 (91)	43.2 (164)
Personal Accomplishment	−0.34	−0.32	34.39 (6.94)	42.6 (162)	30.5 (116)	26.8 (102)

**Table 2:** Burnout in AR professionals.

		Emotional Exhaustion (EE)	Depersonalization (D)	Personal Accomplishment (PA)	Percentage in the High Levels of EE and D, and Low of PA
	n	Mean	Mean	Mean	
EMBRYOLOGISTS	104	25.64	10.25	32.49	EE = 72.1% D = 48.1% Low PA = 48.1
GYNECOLOGISTS	116	24.34	9.11	35.71	EE = 45.7% D = 44.8% Low PA = 28.5%
PSYCHOLOGISTS	31	19.55	6.00	37.61	EE = 32.3% D = 29.0% Low PA = 32.3%
NURSES	74	24.7	8.31	33.80	EE = 44.6% D = 41.9% Low PA = 44.6%
NURSE ASSISTANTS	55	22.11	7.09	34.18	EE = 34.6% D = 32.7% Low PA = 45.5%

**Table 3:** Percentages of stressors experienced by the whole sample and by specific AR professionals.

	Whole Sample	Embryologists	Gynecologists	Psychologists	Nurses	Nurse Assistants
	% (n)	% (n)	% (n)	% (n)	% (n)	% (n)
Working under pressure	76.6 (291)	82.7 (86)	82.8 (96)	45.2 (14)	73.0 (54)	74.5 (41)
Overloaded schedule	54.2 (206)	52.9 (55)	55.2 (64)	45.2 (14)	56.8 (42)	56.4 (31)
Lack of professional recognition	31.1 (118)	17.3 (18)	45.7 (53)	16.1 (5)	39.2 (29)	23.6 (13)
Communication difficulties between the team members	45.0 (171)	49.0 (51)	47.4 (55)	32.3 (10)	43.2 (32)	41.8 (23)
Management of complicated patients	53.2 (202)	61.5 (64)	29.3 (34)	54.8 (17)	71.6 (53)	61.8 (34)
Breaking bad news to patients	31.1 (141)	43.3 (45)	23.3 (27)	41.9 (13)	45.9 (34)	40.0 (22)

**Table 4:** Hierarchical regressions for the three burnout subscales.

		Emotional Exhaustion			Depersonalization			Personal Accomplishment		
		B	SE B	β	B	SE B	β	B	SE B	β
Model 1	Gender	0.81	1.88	0.02	−0.70	0.89	−0.04	0.04	1.01	0.00
	Age	−0.05	0.07	−0.04	−0.07 *	0.03	−0.12	0.10 **	0.04	0.14
	R^2^		0.01			0.02			0.02	
	F for change in R^2^		0.52			2.62			3.97 *	
Model 2	Gender	0.07	1.60	0.02	−0.74	0.78	−0.04	0.36	0.97	0.02
	Age	0.05	0.06	0.04	−0.05	0.03	−0.08	0.07	0.04	0.10
	Working under pressure	12.19 ***	1.41	0.40	3.39 ***	0.77	0.24	−3.14 ***	0.86	−0.19
	Overloaded schedule	0.42	1.21	0.02	−0.29	0.66	−0.02	0.68	0.74	0.05
	Lack of professional recognition	3.10 *	1.24	0.11	1.21	0.67	0.09	−0.55	0.76	−0.04
	Communication difficulties between the team members	3.45 **	1.18	0.13	1.37 *	0.64	0.11	−0.34	0.72	−0.03
	Management of complicated patients	4.37 ***	1.19	0.17	0.73	0.64	0.06	−2.41 ***	0.72	−0.17
	Breaking bad news to patients	3.70 **	1.20	0.14	−0.01	0.65	−0.00	−1.65 *	0.73	−0.12
	R^2^		0.31			0.11			0.12	
	F for change in R^2^		26.78 ***			6.30 ***			6.99 ***	

* = *p* ≤ 0.05; ** = *p* ≤ 0.01; *** = *p* ≤ 0.001.

## Data Availability

The datasets generated and analyzed during the current study are available from the corresponding author upon reasonable request.
